# Immunological Aspects of Approved MS Therapeutics

**DOI:** 10.3389/fimmu.2019.01564

**Published:** 2019-07-11

**Authors:** Paulus S. Rommer, Ron Milo, May H. Han, Sammita Satyanarayan, Johann Sellner, Larissa Hauer, Zsolt Illes, Clemens Warnke, Sarah Laurent, Martin S. Weber, Yinan Zhang, Olaf Stuve

**Affiliations:** ^1^Department of Neurology, Medical University of Vienna, Vienna, Austria; ^2^Department of Neurology, Barzilai University Medical Center, Ashkelon, Israel; ^3^Faculty of Health Sciences, Ben-Gurion University of the Negev, Beer-Sheva, Israel; ^4^Neuroimmunology Division, Department of Neurology and Neurological Sciences, Stanford University, Stanford, CA, United States; ^5^Department of Neurology, Christian Doppler Medical Center, Paracelsus Medical University, Salzburg, Austria; ^6^Department of Neurology, Klinikum Rechts der Isar, Technische Universität, Munich, Germany; ^7^Department of Psychiatry, Psychotherapy, and Psychosomatics, Christian Doppler Medical Center, Paracelsus Medical University, Salzburg, Austria; ^8^Department of Neurology, Odense University Hospital, Odense, Denmark; ^9^Institute of Clinical Research, University of Southern Denmark, Odense, Denmark; ^10^Department of Neurology, Medical Faculty, University of Köln, Cologne, Germany; ^11^Institute of Neuropathology, University Medical Center, Göttingen, Germany; ^12^Department of Neurology, University Medical Center, Göttingen, Germany; ^13^Department of Neurology and Neurotherapeutics, University of Texas Southwestern Medical Center, Dallas, TX, United States; ^14^Neurology Section, VA North Texas Health Care System, Medical Service Dallas, VA Medical Center, Dallas, TX, United States

**Keywords:** multiple sclerosis, immunotherapeutics, immunomodulation, immunosuppression, monoclonal antibodies

## Abstract

Multiple sclerosis (MS) is the most common neurological immune-mediated disease leading to disability in young adults. The outcome of the disease is unpredictable, and over time, neurological disabilities accumulate. Interferon beta-1b was the first drug to be approved in the 1990s for relapsing-remitting MS to modulate the course of the disease. Over the past two decades, the treatment landscape has changed tremendously. Currently, more than a dozen drugs representing 1 substances with different mechanisms of action have been approved (interferon beta preparations, glatiramer acetate, fingolimod, siponimod, mitoxantrone, teriflunomide, dimethyl fumarate, cladribine, alemtuzumab, ocrelizumab, and natalizumab). Ocrelizumab was the first medication to be approved for primary progressive MS. The objective of this review is to present the modes of action of these drugs and their effects on the immunopathogenesis of MS. Each agent's clinical development and potential side effects are discussed.

## Introduction

Multiple sclerosis (MS) is the most common neuroinflammatory and neurodegenerative disease in young adults, with more than 2 million patients worldwide (1). Since the first insights into its pathogenesis were gained from anatomical studies on MS patients in the 19th century by Robert Carswell (2), Jean-Martin Charcot, and others (3), the understanding of pathophysiological concepts concerning MS has been broadened exceedingly. However, modification of the disease course was elusive until the approval of interferon beta-1b (IFN-ß) in 1993 (4). Over the past two decades, the treatment landscape has changed tremendously. Currently, more than a dozen drugs are approved for relapsing-remitting multiple sclerosis (RRMS), and one agent for primary progressive multiple sclerosis (PPMS) (5). These agents represent 10 different substance classes with different modes of action. Whereas, some drugs, including IFN-ß preparations and glatiramer acetate, have no clearly defined mechanisms of action, many of the other agents are target specific and the result of rational drug design. Consequently, much has been learnt about the pathogenesis of MS from the administration of these agents. Although T cells were thought to be the principal lymphocyte subset to initiate and perpetuate disease activity in MS (6), the efficacy of anti-CD20 agents, demonstrated in clinical trials, challenged that concept and pushed B cells together with T cells to the front stage of MS pathogenesis (7, 8).

The initial step in the immune cascade of MS seems to be the activation of T helper (Th) cells in lymph nodes through contact with antigens (either myelin antigens or non-self-antigens sharing similar epitopes to myelin antigens) presented by antigen-presenting cells (APC), including macrophages or dendritic cells (9). This results in the activation and differentiation of myelin-reactive T cells. Activated T cells exit the lymph nodes and circulate in peripheral blood (10), from where they can readily migrate into other tissues, including the central nervous system (CNS). There, these cells can proliferate and clonally expand if they encounter their cognate antigen. The level of chemokines and cytokines increases (e.g., interleukin [IL]-2, IL-1, interferon [IFN]-y, tumor necrosis factor [TNF]-α). As a consequence, additional T and B cells, as well as monocytes are recruited into the CNS and enhance the inflammatory cascade (11).

The monitoring of patients taking new and highly effective drugs that are associated with severe adverse events (AEs) or risks becomes increasingly important. Therefore, it is essential to understand the mode of action of MS drugs and their effects on the immune system. It supports remaining vigilant for unexpected novel AEs that in turn help to understand the mode of action more precisely, and explore the pathophysiology of MS.

This review aims to provide an overview of the approved MS drugs. The history of these drugs and their mode of action are presented considering the current understanding of the pathogenesis of MS.

The review starts with drugs for which the mechanisms of action are not entirely understood, followed by drugs with well-defined molecular and cellular targets.

## Interferon Beta (IFN-ß)

Interferons are part of the cytokine family and are signaling proteins. They can be divided into three classes: type I (interferon alpha and beta), type II (interferon gamma), and type III (interferon delta), with different biological effects (12). IFN-ß belongs to the class of type I interferons and is produced by lymphocytes, fibroblasts, macrophages, and endothelial cells (12). Interferons play an important role in the regulation of the immune system. The effects modulated by IFN-ß are complex and have not been elucidated in detail. IFN-ß binds to the type I IFN receptors INFAR-1 and INFAR-2. Its affinity to INFAR-2 is higher than to INFAR-1. This binding activates the JAK/STAT (janus kinases/signal transducer and activator of transcription proteins) signaling pathway leading to the expression of cellular genes (e.g., Mx protein, ß2-microglobulin, 2′/5′-olygoadenylate synthetase, and neopterin) (13). Overall, the activation of signal-transduction pathways by IFN-ß leads to antiviral, immunomodulatory, and antiproliferative effects (14).

IFN-ß has a wide range of immunomodulatory effects. It reduces the number of dendritic cells and downregulates antigen presentation by APCs in the peripheral blood and also within the CNS (microglial cells and monocytes). The expression of Toll-like receptors (TLR) 3 and 7, as well as MyD88, on dendritic cells is upregulated, leading to altered immune responses. It induces CD4^+^, CD8^+^, CD25^+^, FOXP3^+^, and FoxA1^+^ T cells (Treg cells). IFN-ß decreases inflammatory T cell responses by inhibiting the stimulation and activation of T cells (e.g., by modulating costimulatory molecules on dendritic cells and inhibiting the expression of MHC class II molecules and co-stimulatory factors such as CD80 and CD28 on APCs) (15, 16). The secretion of cytokines and chemokines is altered (e.g., increased levels of IL-10 and IL-4, and decreased levels of IL-12 and TNF α), and the differentiation of CD4+ T cells shifts from a T helper-1 (Th1) to Th2 phenotype, resulting in a less pro-inflammatory and more anti-inflammatory cytokine milieu (17). The number of Th17 cells decreases, leading to a reduction in the release of Il-17 (12), and the apoptosis of auto-reactive T cells is induced (5). Effects on cytokines and chemokines, matrix metalloproteinases (MMP), and adhesion molecules (e.g., VLA-4 on T cells) have been suggested (15, 18–20), thus the migration of leukocytes into the CNS via the blood-brain-barrier (BBB) is reduced.

Currently, IFN-ß is available as IFN-ß-1a (Avonex®, Rebif®, and Plegridy®) and IFN-ß-1b (Betaferon® or Extavia®). IFN-ß-1-a differs from IFN-ß-1b in its amino-acid sequence, tertiary structure, and glycosylation status (21). IFN-ß-1b was the first drug approved by the US Food and Drug Administration (FDA) for the treatment of MS in 1993 (22) and was granted market authorization in 1995 in Europe (23). The preparations differ with respect to their frequency and route of administration. The frequency of administration ranges from every other day/thrice a week (tiw) (Betaferon® or Extavia®/Rebif® subcutaneously [SC]), to once a week (Avonex®, intramuscular [IM]), to biweekly (Plegridy®, SC). PEGylation led to more stable preparations and a longer half-life (24).

When tested in patients with MS, IFN-ß-1b significantly lowered relapse rates in RRMS by approximately one third (25), with more patients free of relapses after 2 years in the IFN-ß-1b cohort (26). No significant differences in disease progression or the relapse rate were confirmed at 6 months in patients with mild (Expanded Disability Status Scale [EDSS] ≤3.5) or moderate (EDSS >3.5) disability (27). In patients with clinically isolated syndrome (CIS) treated with IFN-ß, the conversion rate to MS was lower during the study period compared to the control group (28–30). However, in secondary progressive MS (SPMS), conflicting results were reported between a European (31) and a North American study (32), with positive effects on progression confirmed at 3 months in the European study, which were explained partly by the younger and clinically more active patient population in the European study than the North American study (33). A prospective study of 2,570 IFN-ß-treated MS patients revealed that an early start within the first years after diagnosis significantly lowered the risk of EDSS progression and long term disability (milestone: EDSS 4) (34). A 16-year follow up study of pivotal trials of IFN-ß-1b revealed different mortality rates for the study groups in the pivotal studies, with the highest mortality rate for the cohort initially treated with a placebo (18.3%), followed by the study group given 50 μg every other day (8.3%), and was lowest mortality rate was found in the group given the high and subsequently approved dose of 250 μg (5.4%). Standard long-term assessments did not show differences between the study groups except for mortality (35).

Since IFN-ß is immunogenic, allergic reactions might occur, and importantly, neutralizing antibodies (NAbs) can be formed in response to treatment. NAbs can lead to the decreased efficacy of IFN-ß preparations and a worsening disease outcome might be observed (36). NAbs were more frequently reported during treatment with IFN-ß-1b than with IFN-ß-1a. Based on the data from various trials, IFN-ß-1b seems to be more immunogenic than IFN-ß-1a (4, 37). This was confirmed by samples from 20,695 MS patients from 6 European Countries. IM administered IFN-ß-1a is the least immunogenic IFN-ß preparation followed by SC administered IFN-ß-1a preparations, with SC IFN-ß-1b preparations being the most immunogenic ones (37). The reason for this has not been elucidated.

The most common AEs include influenza-like symptoms, injection-site reactions, headache, thyroid disorders including autoimmunity, depression, allergic reactions, and elevated liver enzymes with the possibility of severe hepatic injury, which are all frequently reported. Hematological abnormalities (leukopenia, lymphopenia) can also be found (4, 5, 23, 26, 27).

Monitoring requirements include blood counts, liver-enzyme assessments and thyroid testing at regular intervals. NAbs should be tested when treatment failure is suspected (5). According to preclinical studies, harm to the fetus cannot be excluded. The data on IFN-ß during pregnancy in MS patients has so far revealed no association between treatment and an increased teratogenic or abortive potential. The data on treatment during the second and third trimester is limited (38). Table 1 shows data on all approved injectables.

**Table 1:** Brand name as well as data on efficacy, dose, route of administration, adverse events of approved injectables.

**DMT**	**Interferon β-1b**	**Interferon β-1a**	**Interferon β-1a**	**Peg-Interferon β-1a**	**Glatiramer acetate**
**Brand name**	**Betaferon^**®**^/Betaseron^**®**^** **Extavia^**®**^**	**Avonex^**®**^**	**Rebif^**®**^**	**Plegridy^**®**^**	**Copaxone^**®**^**
Production process	*E. coli*	Chinese hamster ovary	Chinese hamster ovary	Chinese hamster ovary + Pegylation	Synthetic polymer
Molecular structure	165 AA recombinant Non-glycosylated protein lacking amino acid at position 1, serine substitution for cysteine at position 17	166 AA recombinant glycoprotein Identical to human IFN-β	166 AA recombinant glycoprotein Identical to human IFN-β	166 AA recombinant glycoprotein Identical to human IFN-β + Polyethylene glycol	Random copolymer of glutamate, lysine, alanine, tyrosine
Route	SC	IM	SC	SC	SC
Dose	250 μg	30 μg	22/44 μg	125 μg	20/40 mg
Frequency	Every other day	Weekly	Thrice weekly	Every 2 weeks	Daily/thrice weekly
Study	IFNβ MS Study Group 1993	MSCSG 1996	PRISMS 1998	ADVANCE 2014	Cop1 MSSG 1995/GALA 2013
**Relapses**	0.84	0.61 (ITT −0.67)	0.91/0.86	0.3	0.59/0.33
Annualized rate					
Relative RR	**34%**	**32% (ITT** **−18%)**	**27/33%**	**36%**	**29/34.4%**
Absolute RR	0.43	0.29 (ITT −0.15)	0.37/0.42	0.14	0.25/0.17
NNT	**2.3**	**3.5** (ITT—**6.7**)	**2.7/2.4**	**7**	**4/5.9**
**Reduction in disease**	29%^*^	37%	30%	38%	12%^*^
Progression					
NNT	**9**	**8**	**8**	**37**	**33**
Reduction in new T2	83%	52%	78%	67%	35/35%
and Gd+ MRI activity	75%	50%	84%	86%	39/45%
Main AE	ISR, flu-like symptoms, increased spasticity and fatigue, depression, migraine headache, menstrual irregularities, leukopenia, LFT abnormalities, Thrombotic microangiopathy (manifest mainly as TTP or HUS)				ISR, IPIR, urticaria lipoatrophy, lymphadenopathy

*AA, amino acids; HUS, haemolytic-uremic syndrome; IM, intramuscular; IPIR, immediate post injection reactions; ISR, injection site reactions; ITT, intention to treat; LFT, liver function tests; μg, microgram; MIU, million international units; MRI, magnetic resonance imaging; ND, not determined; SC, subcutaneous; TTP, thrombotic thrombocytopenic purpura*;

* *Non-significant. Bold values indicate most important outcome parameters and AEs*.

## Glatiramer Acetate

Glatiramer acetate (GA, Copaxone®, formerly known as copolymer-1 or Cop-1, and Glatopa®, a biosimilar) is a mixture of random synthetic polypeptides composed of 4 amino acids (glutamate, lysine, alanine, and tyrosine) in a pre-defined molar ratio. GA initially was developed at the Weizmann Institute in Israel as a chemical and immunological analog of the major myelin antigen, myelin basic protein (MBP), to induce experimental autoimmune encephalomyelitis (EAE). Surprisingly, GA did not prove to be encephalitogenic, nor did it induce EAE in susceptible animals, but rather showed high efficacy in suppressing, and even preventing EAE induced by MBP and other myelin antigens in a variety of species and models of EAE (39).

GA's exact mode of action in MS is not completely understood, but extensive research has shown that GA, initially considered to be specific for MBP-related T cell immune responses, affects a variety of immune and non-immune pathways. GA cross-reacts with MBP on the cellular and humoral levels (39) and probably functions as an altered peptide ligand that promotes regulatory T cells instead of stimulating adverse T cell autoreactivity (40). GA's immunomodulatory effects probably stem from strong and indiscriminate binding to MHC class II molecules on APCs, while competing with MBP (41) and probably other myelin antigens (42) for these binding sites. This binding effectively displaces MBP, proteolipid protein (PLP), and MOG-derived peptides from their MHC binding sites (43), resulting in altered T cell responses, as evidenced by the suppression of myelin-reactive T cells by GA (42, 44, 45) and the generation of regulatory Th2 cells recognizing both GA and MBP that can cross the BBB, secrete anti-inflammatory cytokines, and exert bystander suppression of auto-aggressive inflammatory T cells in the CNS (46–48). These GA-specific Th2 cells also secrete large amounts of brain-derived neurotrophic factor (BDNF) that might be neuroprotective (49). Other effects of GA on T cells include T cell receptor (TCR) antagonism via the specific engagement of TCR recognizing MBP through the GA-MHC complex in a manner that results in functional receptor inactivation (50) and induction of regulatory CD4+CD25+ T cells through activation of the transcription factor FoxP3 (51).

GA also modulates macrophages, microglia, and dendritic cells, and drives them into M2 phenotype and anti-inflammatory responses (52–54). Incubation of the human monocytic cell line THP-1 with GA results in down-regulation of the expression of MHC class II and molecules on the cell surface and reduced secretion of TNF-α and cathepsin-B (55). These effects may contribute to the modulation of CNS neuroinflammation (56).

Much attention has recently been drawn to the role of B cells in the pathogenesis of MS, and to the beneficial effects of anti-B cell therapy in both RRMS and PPMS (57). Several recent studies have shown that treatment with GA is associated with reductions in the number of B cells, plasmablasts, and memory B cells, as well as a shift from a pro-inflammatory to an anti-inflammatory B cell phenotype (58). It has been hypothesized that this may be mediated by the cross-reactivity of B cell receptors for GA with antigen (possibly myelin basic protein) expressed in the MS lesion (58).

GA has also been shown to promote repair mechanisms, remyelination, and neurogenesis in the EAE model by augmenting the proliferation, migration, and differentiation of oligodendroglial and neuronal progenitor cells (48, 59).

GA was initially tested on a small number of patients with advanced MS (*n* = 4) or acute disseminated encephalomyelitis (*n* = 3) in Israel (60) and in 16 MS patients in the US (61). No side effects or clinical deterioration were noted, and several patients even improved. These encouraging results prompted a pivotal phase-II clinical trial in 50 RRMS patients who were randomized to receive either daily SC injections of 20 mg GA or matching placebos. A marked reduction in the rate of relapses was noted in the GA group, especially in less-disabled patients (62). However, another 2-center randomized trial in 106 progressive MS patients failed to demonstrate a beneficial effect on disability progression resulting from 15 mg of GA injected SC twice daily. Nevertheless, two additional secondary disability endpoints and the primary endpoint in one center were met (63).

A pivotal phase-III clinical trial with GA was conducted in 11 US centers. In this trial, 251 RRMS patients were randomized to receive either 20 mg of GA or a placebo via daily SC injections for 2 years. A significant 29% reduction in the annual relapse rate (ARR) was observed in the GA group compared to the placebo group (*p* = 0.007). Significantly more patients on GA improved on the EDSS score, and significantly fewer patients worsened (64, 65). Unfortunately, no MRI scans were performed in this trial, except for at one center where patients on GA had significantly fewer gadolinium (Gd)-enhancing lesions and reduced brain volume loss compared to patients taking placebo (66). To better appreciate GA's effect on MRI parameters, 239 RRMS patients in Europe and Canada were randomized to daily GA or placebo treatment and had monthly MRI scans for 9 months. GA reduced the number of Gd-enhancing and new T2-weighted lesions (67) and the proportion of new Gd-enhancing lesions evolving into black holes (68). The daily dose of 20 mg of GA had similar efficacy as 40 mg GA administered daily (69) or thrice weekly (70), and both regimens (20 mg qd or 40 mg tiw) are approved for use in RRMS. Similarly to the interferons, GA has not been shown to reduce disability progression in PPMS (71), but is highly effective in delaying clinically definite MS after CIS (72). Long-term follow-up of patients with RRMS shows continuous efficacy with low relapse rates and minimal EDSS progression after 15 years (73).

In comparative trials with available interferons in RRMS, GA was as effective as IFN-β-1b (74) or SC IFN-β-1a (75), and superior to IM IFN-β-1a (76). The latter study also showed that the combination of GA and IM IFN-β-1a was not superior to either therapy alone (76).

GA's good safety profile has been established over many years of clinical use. Its principal side effects include local-injection-site reactions (tenderness, pruritus, erythema, or induration). Regional lymphadenopathy; local lipoatrophy, which may be permanent; allergic reactions and rare injection site skin necrosis may also occur. About 16% of patients experience a rare systemic post injection reaction comprising of various combinations of the following effects: chest tightness, dyspnea, flushing, palpitations, diaphoresis, and anxiety beginning immediately after GA injection and resolving spontaneously within a few minutes without any sequelae. Unlike IFN-β, treatment with GA is not associated with leukopenia, liver, or thyroid abnormalities; depression; or any additional systemic side effects. It is not associated with any serious AEs seen with other potent newer therapies for MS either, such as opportunistic infections, malignancy, or secondary autoimmunity. Virtually all patients develop binding antibodies, but not NAbs to GA, which do not impair its clinical efficacy (77). GA elicits no adverse effects on fertility, pregnancy, or fetal outcomes (78) and is the only MS drug that is no longer contraindicated during pregnancy in Europe.

Although only moderately effective in reducing disease activity, GA is registered worldwide as a first line platform therapy for patients with RRMS due to its long-term efficacy and safety.

## Fingolimod

Therapeutic concepts in MS include the down-regulation or depletion of pro-inflammatory T and B cells, the enhancement of anti-inflammatory immune responses (79, 80), the prevention of encephalitogenic lymphocytes from entering into the CNS, and the retention of auto-reactive lymphocytes within secondary lymphoid organs (as in the case of fingolimod) (81, 82). This recognition was based on the understanding of the interaction between sphingosine-1-phosphate (S1P), a signaling sphingolipid, and its receptors, S1PR1-5, essential for lymphocytes to egress from secondary lymphoid organs into the systemic circulation (83, 84). The search for molecules targeting the S1P pathway resulted in the discovery of the fungal metabolite myriocin, which eventually led to the development of FTY720 (fingolimod), an oral therapy for treating RRMS (85). FTY720, a functional antagonist of S1PR1-3,4,5 (S1PR1 being the dominant receptor in lymphocytes) (10) binds to the receptor, leading to internalization of the S1P/S1PR complex via the β-arrestin-mediated mechanism (86), thereby preventing lymphocytes' egress (10). This effect is primarily observed in the retention of CD4- and CD8-positive naïve lymphocytes and central memory (CD45RA^−^) T cells in the lymphoid organs. However, effector memory T cells (CD45 RA^+/−^), which primarily use a chemokine-based signaling mechanism, are spared (85, 87). Research dedicated to understanding the effect of fingolimod on lymphocyte subsets additionally identified CD4^+^CD25^+^ regulatory T cell populations as being up-regulated *in-vitro*, which could have implications in down-regulating pro-inflammatory lymphocyte reactivity (88). B lymphocytes are also sequestered in the spleen due to their unique dependence on the S1P pathway, although S1P alone is not sufficient for B cells to exit from the bone marrow (83). S1PRs are expressed at varying levels on endothelial cells, neurons, and CNS glia, however, their function and response to S1PR modulator therapies beyond the immune system are not well-understood (89, 90).

Two landmark, double-blind, randomized trials established the efficacy of fingolimod compared to a placebo or active comparator in treating RRMS. The FREEDOMS trial showed a decrease in the annualized relapse rate (ARR) (0.16 with a regimen of 1.25 mg daily, compared to 0.40 with a placebo), with a relative reduction of ~50% (91). Radiographically, a reduction in both new enhancing and non-enhancing lesions was reported. The results for FREEDOMS II were comparable (92). Subsequently, the TRANSFORMS trial compared two doses of oral fingolimod (0.5 mg daily and 1.25 mg daily) to weekly IM IFN-β-1a for 1 year, which showed a decrease in ARR to 0.16 with 0.5 mg fingolimod, 0.2 with 1.25 mg fingolimod, and 0.33 with IFNβ-1a therapy. Interestingly, this study did not show any difference in the progression of disability assessed using the EDSS (which might be partly explained by the trial design with a short trial duration) (93). The FREEDOMS trial also showed a reduction in the rate of whole-brain atrophy compared to the placebo, suggesting a potential neuro-protective effect of fingolimod (91). However, a recent trial with fingolimod showed no benefit on disability progression compared to placebo when tested for a primary composite endpoint, including EDSS, a 25-foot timed walk test, and a nine-hole peg test in PPMS; although a decrease in radiographic activity has been observed (86). Based on the trial results in RRMS, 0.5 mg daily fingolimod has been approved for the treatment of MS. Fingolimod has been tested in pediatric MS and was associated with a lower rate of relapses and lower accumulation of MRI lesions compared to patients treated with IFN-ß-1a (94). Based on these studies, it has been approved for the treatment of pediatric MS (95)^1^.

Fingolimod's most common AEs include bradycardia and less commonly first/second-degree atrioventricular block, likely due to effects on S1PRs in atrial myocytes (85). Notably, these are often asymptomatic, observed during the administration of the first dose, and they also might recur after an interruption of more than 2 weeks^2^ (96). Macular edema has been shown to occur in <1% of patients during the first 3 months of treatment and often resolves after treatment is discontinued. Disseminated varicella zoster infection occurred in one patient in previous clinical trials. Elevated liver enzymes (>3× upper limit of normal) were also observed in the FREEDOMS trial, though no cases indicated hepatotoxicity (92). Increased rates of lower respiratory tract infections, cutaneous malignancies (not only basal cell carcinoma, but also squamous cell carcinoma and cutaneous lymphoproliferative disorders), and opportunistic infections including cases of progressive multifocal leukoencephalopathy (PML), varicella-zoster-virus (VZV), and herpes-simplex-virus (HSV) associated encephalitis as well as cryptococcal infections have been reported (5, 97). Retrospective reviews of fingolimod's effects on pregnancy from clinical development trials and additional reports from smaller trials have shown few adverse fetal outcomes. However, the number of adverse outcomes and elective abortion were in the expected range of the general population. Data on fingolimod exposure beyond the first trimester is scarce (38). Since fingolimod exposure causes teratogenicity in rodents, a teratogenic potential cannot be ruled out (85). Similarly, since fingolimod can be detected in breast milk, it is also contraindicated in lactating women.

Based on potential untoward effects, screening before initiation of fingolimod treatment comprises baseline laboratory parameters (including a complete blood count, liver-function test, and varicella zoster antibody titers), electrocardiogram, spirometry (in cases of a previous respiratory disease, such as asthma), and an ophthalmologic examination to evaluate for macular edema. Patients are monitored closely for at least 6 h after the first dose (or at re-introduction) in a clinical setting for bradycardia and other cardiac-rhythm abnormalities. Patients with pre-existing cardiac abnormalities, such as conduction block or ischemic heart disease, or those taking medications that interfere with cardiac rhythm and conduction are advised to have a cardiology consultation if clinically indicated (92). Slight and mostly transient hypertension after initial doses of fingolimod also was observed in the FREEDOMS extension study, however, if it did not resolve, it remained stable over the treatment course (91, 98). Increased frequency of basal-cell carcinoma was reported in patients on long-term fingolimod therapy (91). Subsequently, periodic monitoring of blood counts is recommended given lymphopenia's association with fingolimod. The cessation of fingolimod treatment has been associated with cases of severe rebound syndrome leading to severe relapses or high MRI activity. Discontinuing MS treatments needs to be monitored and the sequence of the most suitable treatments needs to be assessed and planned (99).

Recent trials have investigated more receptor-specific agents targeting S1PRsin the hopes of mitigating side effects. Cardiac effects, lymphopenia, elevated liver enzymes, and macular edema still occur with these agents, though a dose-titration strategy was observed to diminish first dose-associated cardiac effects (100). Recently, a remarkable future path for SPMS treatment was revealed in the EXPAND trial, which showed that siponimod (a selective S1P1/S1P5 binding agent) was the first medication of utility in preventing disability progression at 3 months in SPMS (100). It has been approved as Mayzent© by the FDA for CIS, RRMS and active SPMS^3^.

Table 2 shows data on all approved oral drugs.

**Table 2:** Brand name as well as data on efficacy, dose, route of administration, adverse events of approved oral agents.

	**Fingolimod**	**Teriflunomide**	**Dimethyl Fumarate**	**Cladribine**	**Siponimod^+^**
**Brand name**	**Gilenya^**®**^**	**Aubagio^**®**^**	**Tecfidera^**®**^**	**Mavenclad^**®**^**	**Mayzent^**®**^**
Year approved	2010	2012	2013	2017	2019
Target	S1P receptors	DHODH	Nrf2	Purines	S1P1,5 receptors
Dose	0.5 mg	14 mg	240 mg	3.5 mg/kg	2 mg
Frequency	Daily	Daily	Twice daily	Yearly course x 2	Daily
Study	FREEDOMS 2010	TEMSO 2011	DEFINE 2012	CLARITY 2010	EXPAND 2018
**Relapses**					
Annualized rate	0.18	0.37	0.17	0.14	
Relative RR	**54%**	**31%**	**53%**	**57.6%**	**55.5%**
Absolute RR	0.22	0.17	0.19	0.19	
NNT−2y	**4.5**	**5.8**	**5**	**5**	
Disability progression Relative RR	32%	30%	38%	**33%**	**21%**
Absolute RR	0.064	0.071	0.11	0.063	0.055
NNT−2y	**15**	**14**	**9**	**16**	**18**
Reduction in new T2 MRI lesions	74%	67%	85%	73%	79%
Reduction in Gd^+^ MRI lesions	82%	80%	90%	86%	
Reduction in Brain Volume Loss	36%	25%^*^ (BPF) 37% (SIENA)	NA		23.4%
NEDA-3 vs. comparator	33/13%	23/14%	23/11%	47/17%	
Main AE and AE of interest	Bradycardia, AVB, LFT↑, BP↑, Lymphopenia, macular edema, infections, opportunistic infections (**PML**, cryptococcus), skin malignancies	Diarrhea, BP↑, LFT↑ alopecia, PN, Lymphopenia	flushing, GIT symptoms, LFT↑, UTI, Lymphopenia, **PML**	Infections (herpes), lymphopenia, headache, neoplasms? GIT symptoms	Similar to Fingolimod. Less early bradycardia

+ *Not approved by the EMA*.

*AVB, atrio-ventricular block; BP, blood pressure; BPF, brain parenchymal fraction; DHODH, dihydroorotate dehydrogenase; GIT, gastrointestinal tract; LFT, liver function tests; NEDA, no evidence of disease activity (NEDA-3); Nrf2, nuclear factor (erythroid-derived 2)-like 2; PML, progressive multifocal leukoencephalopathy; PN, polyneuropathy; S1P, sphingosine-1-phosphate; SIENA, structural image evaluation, using normalization of atrophy; UTI, urinary tract infection*;

* *Non-significant. Bold values indicate most important outcome parameters and AEs*.

## Mitoxantrone

Mitoxantrone is a synthetic anthracenedione derivate that initially was developed as a cytotoxic treatment for acute myeloid leukemia (101). As a type II topoisomerase inhibitor, the substance has potent anti-inflammatory and, to a lesser extent, immunomodulatory properties (102). The immunosuppressive effect is mediated by effects on proliferating B and T lymphocytes: induction of cell lysis and initiation of programmed cell death (103, 104). Mitoxantrone also demonstrates immunomodulatory effects and preferentially decreases the migratory capacity of monocytes into the CNS and enhances Th2 cytokine production in CD4+T cells (105). *In-vitro*, mitoxantrone interferes with antigen-presenting capabilities of dendritic cells (106).

Mitoxantrone was the first drug that the FDA and several European countries approved to treat worsening relapsing-remitting, secondary-progressive, and progressive-relapsing MS. Research evidence was generated from a phase-III clinical trial in Europe (107) and an earlier phase-II study (108). Both trials confirmed a significant reduction in the relapse rate and worsening of disability. Mitoxantrone is given intravenously (IV) at a dose of 12 mg/m^2^ at 3-month intervals. Some healthcare facilities prefer a fixed-dose regimen of mitoxantrone: 20 mg IV monthly together with methylprednisolone (1 g) (108).

Mitoxantrone has myelotoxic effects and reduces leukocyte counts; thus, its administration is not recommended when neutrophil numbers are below 1,500 mm^3^ (109). Reversible bone-marrow suppression and nausea are common side effects associated with mitoxantrone infusion (110). Dose adjustment based on leukocyte nadir is mandatory to minimize risks for infections, particularly of the urinary tract. Anemia occurred in 15% of patients (grade ≥ 1) (111). Amenorrhea was reported in up to 26% of mitoxantrone-treated women before the age of 45 (112, 113). Liver toxicity and alopecia have also been observed (111).

Severe AEs include therapy-related acute leukemia (TRAL), cardiotoxicity, and colon cancer (114). Acute promyelocytic leukemia (APL) is the most commonly seen TRAL after initiation of mitoxantrone treatment and is characterized by an aggressive, often fulminant disease course due to a life-threatening coagulopathy, e.g., CNS hemorrhages (115). A recent meta-analysis reveals a TRAL risk of ~0.81%, more than 10-fold higher than in previously reported meta-analyses (0.07%) (116). TRAL, in a mitoxantrone setting, has a mortality rate of ~40% (117).

Cardiotoxicity risk increases with cumulative doses of mitoxantrone (118). Therefore, the maximum cumulative dose is restricted to 100–140 mg/m^2^, however, cardiotoxicity can develop after doses well below the current maximum recommended levels (111). Re-evaluation by the European Medicine Agency (EMA) concluded that the ordinarily cumulative life-time doses for MS patients should not exceed 72 mg/m^2^ ^4^. Systolic dysfunction occurs in approximately 12% and congestive heart failure in around 0.4% of treated patients (117). The authors of some studies, therefore, even suggest to limit mitoxantrone treatment to 1 year, or a cumulative dose to <60 mg/m^2^, to reduce the risk of TRAL and cardiotoxicity (119).

Treatment with mitoxantrone requires monitoring for possible cardiotoxicity and APL. Cardiac monitoring via regular echocardiography (measurement of left ventricular ejection fraction [LVEF]) is required before treatment begins, prior to each dose, and annually after the discontinuation of therapy (110). Close monitoring of full blood counts in patients with MS before and after mitoxantrone administration needs to be carried out to monitor leukocyte nadir (mostly after 10–14 days) and a return to normal levels (~21 days). Clinical vigilance and repeated full blood counts are necessary for 5 years after the termination of treatment (116). Mitoxantrone is contraindicated during pregnancy (38).

In recent years, the use of mitoxantrone has decreased due to the risk of severe AEs and the introduction of novel therapies. The agent should be restricted to selected patients with highly active relapsing multiple sclerosis associated with rapidly evolving disability for whom no alternative treatments are available^5^. In addition, clinical and laboratory vigilance is required both during and after mitoxantrone regimens.

## Teriflunomide

Teriflunomide is the active metabolite of leflunomide, which has been used in the treatment of rheumatoid arthritis since 1988. Teriflunomide received approval for treating RRMS in 2012 in the US (7 and 14 mg daily) and in 2013 in Europe (14 mg daily) (120). Teriflunomide interferes with *de-novo* pyrimidine synthesis and DNA replication of highly proliferating T and B cells by reversibly inhibiting the mitochondrial enzyme dihydroorotate dehydrogenase (DHODH). Since resting T cells use nucleotides from degrading DNA and RNA and do not need DHODH, the protective immune responses are maintained, while the proliferation of activated T and B cells is reduced; thus, the viability of immune-cells is not affected. In the Teri-DYNAMIC study, a shift to regulatory T cell subtypes and a reduction in clonal diversity in the CD4^+^T cell repertoire were observed (121). An increase in Treg cells in gut associated lymphoid tissue also characterized protection in the autoimmune inflammatory model of MS (122). Teriflunomide crosses the blood-brain barrier (BBB) (121), decreases microglia proliferation, and induces IL-10 production by microglia *in-vitro* (123). Besides the anti-proliferative effect, leflunomide and potentially teriflunomide, inhibit the production of IL-17, TNF-alpha, protein tyrosine kinases, the NF-kB pathway, and the IgG secretion of activated B cells, and interfere with the kynurenine pathway (120, 124, 125).Teriflunomide induces apoptosis of EBV-transformed B cells (126). In a virus-induced animal model of MS, teriflunomide reduced glutamate levels and excitotoxicity (127). Teriflunomide also promotes oligodendrocyte differentiation *in-vitro*, ameliorated axonopathy by attenuating CD8^+^ T cell cytotoxicity and supported the proliferation of regulatory CD8^+^ T cells in the CNS of mice (128, 129). Despite these potent immunomodulatory and cytostatic effects, protective immune responses against foreign antigens are maintained. In the TERIVA study, more than 90% of the MS patients treated with teriflunomide achieved sufficient seroprotection rates when vaccinated against seasonal influenza (130).

Teriflunomide is administered as an oral drug once daily, and a steady-state concentration is reached after ~3 months. After withdrawal, serum levels are maintained above 0.02 μg/mL for 8 months, and in some individuals, up to 2 years, due to enterohepatic recirculation. Accelerated elimination can be achieved by administering 8 g of oral cholestyramine three times daily for 11 days, which can be reduced to 4 g three times daily in cases of intolerance. Alternatively, 50 g of activated charcoal powder every 12 h for 11 days can be used. To confirm proper elimination, the concentration should be below 0.02 μg/mL in two serum samples obtained 14 days apart (131).

Teriflunomide's efficacy and safety have been investigated extensively in one phase-II (132) and four phase-III (TEMSO, TOWER, TENERE, and TOPIC) clinical trials, all with long-term follow-up data involving several thousand patients (133–136). Over 90% of patients in the TEMSO and TOWER trials had RRMS (133, 134). Patients with a first single clinical episode were enrolled in the TOPIC trial (136). In the TENERE trial, teriflunomide was compared to SC IFN-ß-1a (135), whereas the other three phase-III trials were placebo-controlled.

In these clinical trials, teriflunomide showed a consistent effect on disease activity, measured by its impact on relapses, disability worsening, MRI outcomes, and combined measures such as no evidence of disease activity (NEDA). Compared to the placebo, 14 mg of teriflunomide daily reduced the ARR by 31–36% in the pivotal trials (133–136). Disability progression, confirmed after 3 months, was also reduced significantly by 29.8 and 31.5% in the 14 mg trial group in TOWER and TEMSO studies (133, 134). Similar efficacy data have been observed in real-life settings for up to 28 months (137). Comparison of the pooled phase-III trial data from 14 mg of teriflunomide and dimethyl-fumarate (DMF) (TEMSO/TOWER vs. DEFINE/CONFIRM) revealed similar numbers needed to treat (NNT) to prevent one relapse or worsening disability (121, 138). However, in a recent registry-based study, the ARR was ~49% lower in patients treated with DMF, and teriflunomide treatment was associated with an increased risk of first relapse and increased incidence of discontinuation due to disease breakthrough (139). In another recent registry-based study with large patient populations, the ARR was similar between teriflunomide and DMF, and discontinuation rate was also similar; nevertheless, ARR were lower in patients treated with fingolimod compared to both DMF and teriflunomide, while disability accumulation was the same (140). A recent Italian study did not observe differences in discontinuation either during the first 24 months (141). In the TEMSO study, the 14 mg daily dosage reduced the number of Gd-enhancing lesions by 80.4% and the total lesion volume by 67.4%; the effect of a 7 mg daily dose was less but still significant (133). The treatment (14 mg) of patients with a first clinical episode suggestive of MS, i.e., CIS, reduced the risk of conversion to clinically definite MS by 42.6%, however, only 44% of the patients completed the study due to early termination related to changes in MS diagnostic criteria (136).

The long-term outcomes in extension studies indicate that the effect of teriflunomide is maintained (class-III evidence), however, the dropout rates varied, ranging from 40 to 75% (121). The analysis of pooled TEMSO/TOWER long-term data (up to 9 and 5.5 years, respectively) indicated that more than 80% of the 122 patients with progressive relapsing MS did not experience worsening disability (121).

The AEs reported more frequently with teriflunomide than the placebo include hair thinning, ALT increase, nausea, diarrhea, paraesthesia, limb pain, arthralgia, nasopharyngitis, polyneuropathy, and menorrhagia. Hair thinning appeared in 10–14% of patients and led to discontinuation in 1.4–2% of cases in pivotal trials (133, 134, 142). The discontinuation of treatment was most commonly related to ALT elevation driven by the trial protocols. In real life, gastrointestinal AE was the most common cause of discontinuation (143). The pooled analysis of safety data from the phase-II, TEMSO, TOWER, TOPIC, extension of phase-II, and TEMSO (up to 12 years), in addition to the safety data from the TOWER, TOPIC, and TENERE extensions (up to 7 years), consistently supported the long-term beneficial AE profile (121, 143, 144). Deaths were not more common in the active arm compared to placebos in pivotal trials, and two deaths in the extension phases (pulmonary tuberculosis and suicide) were potentially related to treatment (121, 133–136, 143–145). A single case of PML after 3 months of teriflunomide treatment has been reported, but it most likely was carried over from preceding natalizumab therapy (146). During the 2.1 million patient years of leflunomide therapy since 1991, two cases of PML have been reported during monotherapy (120).

Teriflunomide is contraindicated during pregnancy and is classified as category X based on embryo-fetal toxicity and malformations in rats and rabbits (121). However, results from animal studies cannot be transferred unrestrictedly to humans, and among 26 reported live births, by human women taking the drug no abnormalities were present (147). The FDA suggests discontinuing teriflunomide in males who wish to father a child, however, this is not required in Europe. Accelerated elimination is necessary for women taking the drug before pregnancy who wish to become pregnant, and serum concentration must be <0.02 μg/mL in two serum samples obtained 14 days apart.

In summary, extensive and long-term data consistently indicate that teriflunomide's efficacy resembles that of injectables, and that it offers a beneficial AE profile. Its administration is convenient, however, frequent blood tests (blood count and liver transaminases) are required during the first 6 months of treatment.

## Dimethyl Fumarate (DMF)

DMF has been used to treat psoriasis since 1959 and was approved to treat RRMS in 2013 (148). It is administered as a 240 mg tablet twice daily.

One of the major mechanisms of DMF and its active metabolite mono-methyl fumarate (MMF) is an antioxidant response through activation of the Nrf-2 pathway, which might result in neuroprotective properties besides influencing NF-κB related cellular responses (149, 150). Activation of the Nrf-2 pathway leads to an expansion of FoxP3^+^ regulatory T cells and CD56^bright^ natural killer cells, as well as to a reduced level of CD8^+^ T cells and B cells (151). DMF yields profound effects on immune responses *in-vitro* and *in-vivo*: It inhibits NF-κB activation and pro-inflammatory cytokine production by myeloid cells; reduces the generation of encephalitogenic T cells, partially by inhibiting antigen presentation; generates a shift from a Th1/Th17 to a Th2 profile; alters cytokine production by B cells; promotes apoptosis of B and T cells; and elicits an anti-proliferative effect (149, 152–156). In MS patients treated with DMF, the T and B cell subpopulations are reduced, and functional changes are observed in lymphocytes and APCs. Such reductions affect mostly cytotoxic T cells, effector/central memory T cells, Th1 cells, Th17 cells, mucosa-associated lymphoid tissue (MALT) cells follicular T cells with a Tfh1/17 phenotype, antigen experienced and memory B cells, and B cells producing TNF. Immunoregulatory CD56^bright^ NK-cells, naïve T and B cells, Th2 cells, FoxP3^+^ Tregs, and follicular T cells with a Tfh2 phenotype are increased (151, 153–155, 157–162). Such a pro-tolerogenic shift is associated with NEDA in MS patients (158); higher levels of the NRF2 target gene NAD(P)H quinone dehydrogenase 1 (NQO1) was also associated with NEDA status after 1 year of DMF treatment (151). MMF crosses the BBB, and DMF/MMF alters the function of CNS resident cells *in-vitro*, suppresses inflammatory cytokine production by activated microglia and astrocytes, and increases the number of oligodendrocyte precursor cells (163–165).

DMF's clinical efficacy and safety as an MS drug have been investigated in two randomized placebo-controlled phase-III trials, DEFINE and CONFIRM (166, 167). An active agent, GA, was also included as a reference comparator in the CONFIRM trial. The ARR was reduced by 53% in the DEFINE study and 44% in the CONFIRM study, compared to the placebo (166, 167). The risk of confirmed disability progression sustained for 12 weeks was also reduced by 38% in the DEFINE study and 21% in the CONFIRM study (166, 167). The integrated analysis of the phase-III trials indicated a 32% (29%) risk reduction in 12 (24)-week confirmed disability progression (168). DMF reduced the number of new or enlarging hyperintense lesions on T2-weighted images by 71 and 85%, respectively, and reduced the odds of an increase in the number of Gd-enhancing lesions by 74% and 90% in the CONFIRM and DEFINE study, respectively (166, 167). Compared to the active comparator GA, DMF twice daily also significantly reduced T2-weighted hyperintense lesions in the CONFIRM trial, whereas the other efficacy outcomes were no different (167). The integrated analysis of CONFIRM and DEFINE demonstrated a 38.9% relative reduction in clinical disease activity (relapse and disability progression) over 2 years compared to placebo treated patients (169).

The phase-III trials, integrated analyses, and follow-up studies all indicated DMF's safety and beneficial AE profile. The frequency of AEs and serious AEs was similar to the placebo in the DEFINE and CONFIRM trials (serious AEs 17 and 18% vs. 21 and 22%, respectively) and GA in the CONFIRM study (17%) (166, 167). The most common AEs were flushing (31–38%), diarrhea (13–15%), nausea (11–13%), upper abdominal pain (10%), and vomiting (10%) (166, 167, 170). Increased liver enzymes were detected in 3–6% of patients treated with DMF (171). Overall, the incidence of AEs leading to discontinuation of the study drug was similar across groups. Discontinuations due to flushing and overall gastrointestinal events occurred more frequently in patients who received DMF (166, 167). Compared to the placebo group, at 1 year, the white-cell and lymphocyte counts decreased by ~10 and 28%, respectively (166). Grade 2 or 3 lymphopenia occurred in 4–10% of the patients compared to 1% or less in the placebo group and tends to persist in some patients (166, 167). Infections were common but the incidence was not significantly different between the DMF and placebo or GA groups (50–68%) (171). Although serious and opportunistic infections were not more common among patients treated with DMF, five cases of PML were reported by 2018 (172). Additional 14 cases were related to other DMF formulas used in psoriasis, and 13 out of the 19 cases had grade 3 lymphopenia (173). CD4^+^ and CD8^+^ T cell repopulation rates are delayed after switching to other disease modifying therapies (DMT) from DMF, and T cell counts may not recover or even continue to decline if DMF treatment is switched to fingolimod or alemtuzumab (174). Decrease of CD8^+^ and memory T cells is more likely compared to CD4^+^ and naïve T cells (156).

In addition to pivotal trials, a few studies have investigated DMF's efficacy and safety in real-life settings, and these recapitulated the findings from the pivotal trials. In two multicenter studies with 1,089 and 735 patients treated for up to 25 and 33 months, the ARR was reduced by 77 and 63% respectively (175, 176), whereas in another multicenter study, the ARR was reduced by 33% (177). NEDA status was achieved in 47.8% of patients after 1 year (176). In the first year, 11–19.5% of patients discontinued treatment, and 30% of patients stopped DMF after 2 years mainly due to poor tolerance (175, 176). Approximately one third of the patients had flushing or gastrointestinal AEs (176). Lymphocytopenia occurred in 16.5 and 18.7% of the patients, respectively (175, 176). Lower baseline lymphocyte counts, female gender and older age (>55 years) were associated with more severe lymphopenia (178). Several recent studies have highlighted the importance of early AE management to improve adherence. In a cohort of 400 patients, 34% stopped treatment within a year and 57% within 2 years (179). The data on treatment with DMF during pregnancy is limited, thus, no final assessment is possible. Generally, it is recommended to stop DMF when planning to conceive (5, 38). Several studies have investigated DMF's efficacy in relation to other DMTs. Fingolimod and DMF were evaluated based on the data from a pivotal study using a matching-adjusted comparison and revealed no significant differences in the effect on clinical parameters between the treatments (180). Similar results were shown in an Italian study based on real-world data. A propensity score-matched study revealed a similar NEDA-3 status for fingolimod (73%) and DMF (70%), however, in patients having switched from other therapeutics, fingolimod was superior to DMF (*p* = 0.007) (181). Another study measuring the indirect effectiveness of fingolimod vs. DMF vs. teriflunomide based on phase-III studies suggested that the probability of achieving NEDA-3 was highest for fingolimod (182). Similarly, a recent study including 3,728 patients from MSBase showed a superior effect of fingolimod on relapse rates and comparable results for disability progression in patients treated with fingolimod, DMF, and teriflunomide (140).

In the STRATEGY study, the risk of relapse after switching from natalizumab was 19.6%, and the ARR was lower in patients with <90 days of a washout period (183). Another study also indicated that DMF can be an option for patients discontinuing natalizumab: After 2 years of DMF treatment, 80% of the patients did not present clinical or MRI evidence of disease activity, and a post natalizumab rebound was observed in 1 out of 39 patients (184).

In summary, the data indicate DMF's efficacy and safety in treating RRMS. Whether its efficacy is higher than teriflunomide's and like fingolimod's is debatable. Sustained lymphopenia after stopping a DMF regimen might complicate the escalation to fingolimod and lymphocyte-depleting therapies. Whereas, pre-treatment with aspirin might mitigate flushing, gastrointestinal side effects are only slighthly mitigated by dose titration and are not worsened by pre-treatment with aspirin (185). Gastrointestinal side effects need early symptomatic treatments which may increase adherence significantly (186).

## Cladribine

Cladribine (2-chloro-2′-deoxyadenosine) is a pro-drug that requires intracellular phosphorylation to become an active purine nucleoside analog that interferes with DNA synthesis and repair, and ultimately leads to cell death. The higher ratio of activity between certain enzymes that activate (desoxycytidine kinase) or deactivate (adenosine-monophosphate kinase and nukleoside-diphosphate kinase) the pro-drug explains the preferential and long-lasting depletion of peripheral B and T lymphocytes with a relative sparing of other hematogenic and immune cells. B and T cells are rapidly depleted. The slight recovery of Tregs before B and T cells repopulation might partly explain the long-lasting effects (187). A parenteral formulation of cladribine was first developed for therapy against hairy-cell leukemia, while the oral formulation of cladribine was developed later and tested in RRMS (188, 189).

Oral cladribine was studied in a phase-III trial (CLARITY) (190), a 96-week, placebo-controlled, double-blind, multicenter study. Patients with active RRMS (at least one relapse within 12 months prior to study entry) were included in the trial. Cladribine was administered based on body weight and tested in three groups: 3.5 mg/kg, 5.25 mg/kg, or a placebo. Compared to the placebo, the ARR at week 96 was reduced in both treatment groups by ~57%. The proportion of patients remaining free of relapses at week 96 increased from ~61 to 80%, resulting in an absolute benefit for approximately 19 out of 100 patients treated. In addition, the relative reduction in the risk of a 3-month sustained progression of disability in both cladribine groups, compared to the placebo group, was 31–33%, and patients treated with cladribine had a reduction of 77% in mean active T2 lesions on MRI (190). Furthermore, in patients with a first clinical attack, cladribine was shown to reduce the risk of a second attack, or three-month EDSS progression (191, 192).

The subsequent CLARITY EXTENSION study showed that treatment with cladribine for 2 years followed by 2 years' placebo treatment produced durable clinical benefits similar to 4 years of cladribine treatment, i.e., approximately 75% of patients treated with cladribine 3.5 mg/kg in CLARITY, remained relapse-free when given placebo during the extension (193, 194).

In the CLARITY study, at a dosage of 3.5 mg/kg, CD4+ T cells dropped by 40–45% and CD8+ T cells by 15–30% without significant recovery prior to the next treatment cycle. CD19+ B cells dropped by ~70–90%, slowly recovering to 15–25% of the baseline (195), suggesting a combined T and B cell-mediated mode of action.

Lymphopenia was dose-dependent (nadir at 4 months), with grade 3 lymphopenia (500–200 cells/uL) in ~25% of patients in the 3.5 mg/kg dose group, and grade 4 (<200 cells/uL) in <1% (194). The rate of common infections was similar when comparing placebo- and cladribine-treated patients. The rate of herpes zoster infections per 100 patient years was higher in the 3.5 mg/kg group than the placebo group (0.83 vs. 0.20) and associated with lymphopenia, explaining why patients with grade 4 lymphopenia should receive a prophylactic anti-herpes infection treatment. Furthermore, the incidence of severe infections was generally higher among patients with lymphopenia and who were taking cladribine at a dosage of 3.5 mg/kg compared to the placebo group (194). PML was not reported during an observational period of >8,500 patient years in the MS indication, whereas some PML cases have been observed with parenteral cladribine in lymphoma patients^6^. Three cases of tuberculosis were reported during the clinical trials, of which one case was fatal. Two cases of hepatitis B occurred, and one of those patients died (166).

Thus, not only clinical follow-up and standard laboratory tests, but also screening for HIV infection, active tuberculosis, and hepatitis are mandatory prior to a treatment course of cladribine. The malignancy rates were higher among cladribine-treated patients compared to the placebo cohort (33 vs. 4); these malignancies comprised of solid tumors with no specific patterns typical of tumors commonly seen during immunosuppression. No cases of leukemia, lymphoma, or lymphoproliferative disorders were reported (166). However, this imbalance explained the initial application rejection by the EMA in 2011, when additional safety data were requested. Such data then were obtained from clinical extension studies, meta-analyses of several other clinical studies with alternative MS drugs, and comparisons with epidemiological data, leading to EU approval in 2017, since the malignancy risk is comparable to other treatment options for MS. However, the EU approved cladribine only for “the treatment of adult patients with highly active relapsing MS as defined by clinical or imaging features”^7^, and it was approved by the FDA in 2019^8^. Furthermore, due to potential teratogenic effects, both males and females must use effective contraception during therapy with cladribine, and for 6 months after a treatment cycle.

Overall, the registered dose of 3.5 mg/kg can be applied orally in short treatment cycles, which might lead to a high adherence to therapy, followed by a sustained therapeutic effect, with efficacy confirmed in highly active patients with the registered indication^7^. The downside of cladribine is that lymphopenia can be severe and frequently reaches grade 3, which is associated with a higher risk of infections. Data on lymphopenia in patients with prior immunosuppressive treatment is lacking (due to exclusion criteria in pivotal trials), thus more data needs to be collected. Furthermore, the long-term risks of malignancy and opportunistic infections remains to be established, as well as algorithms on how to treat patients with ongoing disease activity after a 2-year course of therapy. Finally, cladribine interferes with DNA synthesis and repair mechanisms, raising concerns in young adults of child-bearing age until additional safety data become available.

## Alemtuzumab

Alemtuzumab is a humanized monoclonal IgG1-antibody that targets CD52, a surface molecule with largely unknown functions predominantly expressed at high levels on B and T cells (196, 197). Lower expression levels are found on monocytes, macrophages, and eosinophils. Mature NK cells, plasma cells, neutrophils, and, most importantly, hematological stem cells show little or no expression (198).

Alemtuzumab leads to a rapid and long-lasting depletion of CD52-positive cells by antibody-dependent, cell-mediated cytolysis (ADCC) and complement dependent cytolysis (CDC) (199), followed by a slow repopulation arising from unaffected hematopoietic precursor cells. Both, quantitative and qualitative changes in the immune-cell repertoire are observed, which might contribute to a rebalancing of autoimmune processes. While the exact mechanisms underlying the reprogramming of the immune system are only vaguely understood, a specific pattern exists to repopulate immune-cell subsets in peripheral blood (200, 201). Monocytes reach baseline levels after 3 months. B cell counts not only return to baseline numbers after 3 months, but also show an excess increase to 124–165% of baseline levels at 12 months. CD8+T lymphocytes are restored after 31 months, whereas CD4+T lymphocytes need ~60 months for complete repopulation. This rapid CD19+ B cell subset repopulation in the absence of effective T cell regulation might explain some of the AEs, e.g., secondary autoimmunities (202). Furthermore, an expansion in CD56bright NK cells also occurs (203). The effects of NK cells on disease progression are unclear, however, it is debated whether they will exhibit immunoregulatory properties (204).

Alemtuzumab's efficacy and safety have been evaluated in treatment naive RRMS patients in phase-II (205) and phase-III trials (206), and in RRMS patients who had an inadequate response (≥1 relapse after ≥6 months of treatment) to prior therapy (207). Due to different inclusion criteria, patients in CARE MS I were younger (mean age was 33.0 vs. 34.7 years), had a lower mean EDSS (2.0 vs. 2.7) and a shorter mean disease duration (2.1 vs. 4.5 years). In the phase-III CARE-MS trials, alemtuzumab demonstrated significantly lower disease activity over 2 years vs. SC IFN-ß-1a administered three times per week (206, 207). In both CARE-MS I and II studies, alemtuzumab significantly reduced the frequency of relapses over 2 years compared to SC IFN-ß-1a (54.9 and 49.4% reduction in relapses in the respective trials); significantly improved MRI outcomes including gd-enhancing lesions and new or enlarging T2 lesions in the alemtuzumab cohort compared to the IFN-ß-1a cohort, and significantly reduced the rate of brain-volume loss. Alemtuzumab also significantly reduced the rate of clinical disease worsening over 36 months in the phase-II CAMMS223 study (205). In CARE-MS II, patients treated with alemtuzumab were more likely to experience 6-month confirmed disability improvement than patients receiving SC IFN-ß-1a treatment (hazard ratio 2.57), whereas this outcome was not significant in CARE-MS I (206, 207).

AEs include infusion-associated reactions (IARs), serious infections, and autoimmune-adverse events, including thyroid disorders and, less frequently, immune thrombocytopenia (ITP) and nephropathies. Malignancies such as thyroid cancer, melanoma, and melanoma-*in-situ* as well as lymphoproliferative disorders have been reported^9^.

The IAR rate in the phase-III trials was >90%, mostly mild to moderate in severity and most frequently within the first 3 days of infusion (206, 207). The IARs, which are attributable mainly to cytokine-release syndrome, included headaches, rash, pyrexia, nausea, urticaria, pruritus, flushing, insomnia, fatigue, chills, chest discomfort, and dyspnea. The IARs decrease with successive infusions in a single course and in the second course (206). The clinical trials reported severe IARs ranging from 1 to 3%. Concomitant corticosteroids, antihistamines, and antipyretic drugs are applied with the infusion to avoid IAR. In addition, IARs might be reduced by slowing or temporarily stopping the infusion. Following a safety announcement by the FDA on the rare but serious risks of stroke and blood vessel wall tear (208), Azevedo et al. reported five patients that developed intracerebral hemorrhage within a few hours after administration of alemtuzumab (209).

Moreover, in all patients an increase in blood pressure or labile blood pressure was recognized. Labile hemodynamics under alemtuzumab treatment and infusion-associated reactions resulting in an activated immune system involving mast cells, basophils, complement, activation of platelet derived growth factor, and the release of interleukin-6 (Il-6), or tumor necrosis factor α (TNFα) are discussed as possible causes (210).

Infections are mostly mild to moderate and include oral herpes, herpes zoster, nasopharyngitis, urinary-tract infection, upper respiratory-tract infection, sinusitis, influenza, bronchitis, and localized superficial fungal infections. Serious infections were rare, although slightly elevated with alemtuzumab vs. SC IFN-ß-1a (205–207). Since herpes-virus infections increased in clinical trials with alemtuzumab, prophylactic treatment with an oral anti-herpes agent on the first day of alemtuzumab usage and for 1 month of each treatment cycle was introduced in the risk-management plan. Tuberculosis has been reported in patients treated with alemtuzumab; thus, before the initiation of therapy, all patients must be evaluated for both, active and inactive (“latent”) tuberculosis infection and treated according to local guidelines if required. Moreover, before receiving treatment with alemtuzumab, patients who have not contracted chickenpox and who have not been vaccinated against VZV should be tested for anti-VZV antibodies. Several cases of opportunistic infections including listeria meningitis, esophageal candidiasis, pyogenic granuloma, spirochetal gingivitis, nocardiosis, and cytomegalovirus were reported.

Listeria infections occur generally within 1 month of infusion. Thus, dietary recommendations require the exclusion of certain foods, such as unpasteurized milk and raw meat, during and for 1 month after treatment with alemtuzumab. Antibacterial treatment may be recommended depending on the different regulatory authorities.

Autoimmune AEs represent the most important risk associated with alemtuzumab treatment. These most commonly affect the thyroid; however, they can include rare cases of ITP and anti-glomerular basement membrane nephropathy. The exact pathomechanism leading to secondary autoimmunity remains to be determined. Currently, it is thought that the different temporal lymphocyte repopulation plays a role in this process (211, 212).

In the 5-year follow-up of CAMMS223, thyroid autoimmune AEs occurred in 39% of patients treated with alemtuzumab 12 mg (213). Onset ranged from 6 to 61 months after the first treatment course (207). Incidence peaked at year 3 and declined in subsequent years. Serious ITP events have been observed in ~1% of patients treated with alemtuzumab in the CARE-MS program, between 14 and 36 months after first exposure to alemtuzumab. The first ITP case, during the phase-II CAMMS223 trial, went unrecognized, and the patient died from intracerebral hemorrhage. Following this index case, a monitoring program was implemented to identify and manage ITP systematically, including education on the signs and symptoms for patients and physicians and monthly blood monitoring (142). Other autoimmune cytopenias such as neutropenia, hemolytic anemia, agranulocytosis, and pancytopenia have been reported in the CARE-MS trials with a lower incidence than ITP (206, 207). One patient experienced a recurrence of pancytopenia, which was associated with a lack of compliance with corticosteroid therapy, resulting in fatal sepsis 20 months after alemtuzumab treatment was completed w.

In pilot studies, two patients developed anti-glomerular basement membrane (anti-GBM) disease that ultimately required a kidney transplant (214). In phase-II and phase-III trials, four cases of glomerulonephritis occurred among 1,486 patients treated with alemtuzumab (0.3%). The onset ranged from 4 to 39 months after the last dose of alemtuzumab. Improvements in renal function were observed in two cases of anti-GBM disease after treatment with plasmapheresis, cyclophosphamide, and glucocorticosteroids, and in two cases of membranous glomerulonephritis after treatment with diuretics and/or lisinopril (215, 216).

Secondary autoimmunity is of special interest, since thyroid autoimmunity might affect almost half of the patients (217). A monitoring program was designed and implemented to facilitate the early detection of autoimmune events to ensure timely and adequate management (218): TSH measurements should be performed at baseline and every 3 months for 48 months following the last course (second or subsequent course) (219), and the patient should be monitored for any drug-induced ITP symptoms. Petechiae are uncommon and are usually observed on the lower limbs of patients with a platelet count <20 × 10^9^/L (and often <10 × 10^9^/L). Thus, a platelet count should be performed before the initial course of alemtuzumab, followed by monthly testing that should be continued until 48 months after the final course (220). The signs and symptoms of nephropathy often are non-specific. Routine creatinine testing should be performed before treatment, followed by monthly testing during treatment, continuing until 48 months after the last course (second or subsequent course) (221). Since it is given in cycles, there are no continuous levels of alemtuzumab in the blood. It is recommended that contraception should be used for four months after the last dose (221).

Alemtuzumab's high efficacy contrasts its considerable risks; thus, a thorough assessment of the benefits and risks, adherence to long-term monitoring requirements, and pharmacovigilance are mandatory. Long-term monthly monitoring for 48 months after the final alemtuzumab infusion poses a challenge to patient adherence and requires appropriate education of both physicians and patients. Due to the recently reported side effects including immune-mediated conditions and heart and vessels disorders, the EMA started a review on the medication and temporarily restricted it to patients with highly active disease despite treatment with at least two disease-modifying therapies or in cases when other therapies cannot be used (222). Nevertheless, it is a highly effective treatment option with a long-lasting clinical experience. It should be used in the appropriate patients with the appropriate monitoring schemes.

Table 3 shows data on all approved monoclonal antibodies.

**Table 3:** Brand name as well as data on efficacy, dose, route of administration, adverse events of approved monoclonal antibodies.

	**Natalizumab**	**Alemtuzumab**	**Ocrelizumab**
**Brand name**	**Tysabri^**®**^**	**Lemtrada^**®**^**	**Ocrevus^**®**^**
Year approved	2004, 2006	2013	2017
Target	VLA-4	CD52	CD20
Dose	300 mg	12 mg	600 mg
Route	IV	IV	IV
Frequency	Every 4 weeks	Annual course	Every 6 months
Study	AFFIRM 2006	CARE-MS II 2012 (vs. SC IFNβ-1a)	OPERA/ORATORIO (vs. IM IFNβ-1a/placebo)
**Relapses**			
Annualized rate	0.23	0.26	0.155
Relative RR	**68%**	**50%**	**46%**
Absolute RR	0.5	0.26	0.135
NNT−2y	**2**	**4**	**7.4**
Disability progression			RRMS/PPMS
Relative RR	42%	40%	40/24%
Absolute RR	0.12	0.084	0.054/0.115
NNT−2y	**8**	**12**	**18.5/7**
Reduction in new T2 MRI lesions	83%	32% fewer pt.	80/92%
Reduction in Gd+ MRI lesions	92%	61% fewer pt.	94/95%
Reduction in Brain Volume Loss	NA	23%	19/17.5%
NEDA-3 vs. comparator	37/7%	32/14%	48/27%
Main AE and AE of interest	Infections (**PML**, Herpes), infusion reactions, hepatotoxicity	Infusion reactions, cytopenia, autoimmunity, infections, opportunistic infections, malignancy?	Infusion reactions, Infections

*BBB, blood-brain barrier; IAR, infusion associated reactions; IM, intramuscular; IRR, Injection related reactions; IV, intravenous; NEDA, no evidence of disease activity (NEDA-3); NK, natural killer; SC, subcutaneous; VLA-4, very late antigen-4; PML, progressive multifocal leukoencephalopathy. Bold values indicate most important outcome parameters and AEs*.

## Ocrelizumab

Within the past two decades, the pathogenic role of B cells has generated enormous interest in MS research. Traditionally, MS was primarily considered a T cell-mediated inflammatory disorder, although several findings, including, first and foremost, the development of oligoclonal bands (OCB) in the cerebrospinal fluid (CSF), have indicated a role for B cells. Besides being the source of antibody-producing plasma cells, B cells directly contribute to the development and progression of MS. Peripheral and CNS B cells show signs of chronic inflammation, along with a shift toward antigen-experienced memory B cells (223), indicative of an antigen-mediated activation of B cells in MS. Assumedly, as a consequence, MS patients' B cells show an increased expression of major histocompatibility complex (MHC) class II molecules (224), as well as a higher level of co-stimulatory molecules (225, 226), with the potential to promote the pro-inflammatory differentiation of responding T cells (227). Additional roles of B cells in MS pathogenesis are discussed: antigen presentation, driving T cell activation and auto-proliferation, unbalanced cytokine production, and the formation of ectopic lymphoid follicles (TLOs) under the meninges (228, 229).

Predominantly driven by the assumption that immunoglobulins reactive to a yet unknown self-antigen of the CNS are important drivers of MS pathogenesis, the concept of applying B cell-depleting therapies in MS has evolved. Monoclonal antibodies against CD20 deplete immature and mature B cells, but spare plasma cells and hematopoietic stem cells due to their lack of CD20 expression. Rituximab was the first anti-CD20 antibody to be tested in MS trials, and resulted in a rapid decline in the development of new CNS lesions in patients with RRMS (230, 231). In PPMS, a subgroup of young patients with ongoing CNS lesion formation experienced a slowing of disease progression (232). Testing the humanized successor of rituximab, ocrelizumab confirmed a substantial reduction in the frequency of clinical relapses and CNS lesion formation in RRMS (7, 233). Two identical, randomized, double-blind, double-dummy trials comparing IV ocrelizumab to an active comparator, SC IFN-ß-1a, demonstrated a substantially reduced ARR (0.16 vs. 0.29 *p* < 0.001 in both trials) in patients treated with ocrelizumab (7). Furthermore, ocrelizumab was superior to IFN-ß-1a with respect to disability progression confirmed at 12 and 24 weeks. In addition to these highly promising findings in RRMS, a placebo-controlled, phase-III trial in patients with PPMS revealed a significantly decelerated accumulation of disability, particularly in younger patients with MRI findings suggestive of ongoing inflammatory activity (8). Based on these phase-III clinical-trial findings, ocrelizumab has been approved recently for both MS indications: RRMS and PPMS. IARs have been observed, especially during the first administrations. Neoplasms (including breast carcinomas) were more often reported (2.3%) in the ocrelizumab cohort than in those patients receiving placebo (0.8%) in the PPMS trials (8). Long term effects of immunosuppression and B depletion are missing.

The anti-CD20 monoclonal antibodies rituximab and ocrelizumab differ from each other in certain aspects. Rituximab, which has not been brought to a phase-III trial mainly for strategic considerations, is a chimeric antibody and acts predominantly via CDC. Ocrelizumab is more humanized, and its B cell-depleting effector mechanism is mediated more by ADCC. A third anti-CD20 antibody currently tested in phase-III MS trials is ofatumumab (NCT02792231 and NCT02792218), a fully human anti-CD20 antibody (234).

Ocrelizumab is administered IV every 24 weeks at a maintenance dose of 600 mg. Within this interval, the vast majority of patients are continuously depleted of blood B cells. Very little is known about other bodily systems that might be more important immunologically, such as secondary lymphoid organs. In this regard, a recent experimental study revealed that a fraction of CD20^+^B cells in the spleen is resistant to systemic anti-CD20 treatment (235). After cessation of treatment, this population expanded in parallel to *de novo* B cell generation from bone marrow, resulting in an increased frequency of potentially pathogenic B cells in mice containing a B cell-stimulating immunization. This may be enormously important, since in classical autoimmune diseases, such as myasthenia gravis or AQP4-Ab^+^ NMO, the stimulating autoantigen may be present when B cells return after cessation of anti-CD20 treatment. Furthermore, the extinction and recovery of B cells may differ substantially, both quantitatively and qualitatively, when lower doses or other administration routes are used (236). In accordance with this, studies in rheumatoid-arthritis patients revealed that a single administration of 10 mg of ocrelizumab was sufficient to deplete B cells efficiently from blood, whereas B cell recovery started much earlier than with higher doses (237). Along with these lines, investigations have recently suggested that substantially lower doses of SC ofatumumab are sufficient to mediate a virtually complete removal of B cells from the blood (234, 238). Regarding other compartments, such as secondary lymphoid organs, experimental studies have suggested that a SC administration targets B cells most efficiently in draining lymph nodes, whereas the IV application of anti-CD20 exerts a more thorough effect on the removal of splenic B cells (238). These differences might have important clinical implications, since they might substantially impact both the clinical efficacy and safety of anti-CD20 treatment in MS patients. In pivotal trials, 12 patients developed anti-drug antibodies, and two of these were positive for neutralizing antibodies. Due to the low number and low incidence of these antibodies, no final assessment on their incidence and their impact possible^10^.

Data on administration of ocrelizumab during pregnancy is scarce, thus no final assessment is possible (239)^10^^,^^11^. However, the FDA (the EMA) requires 6 (12) months contraception after the last dose of ocrelizumab.

## Natalizumab

A hallmark in the pathogenesis of MS is immune dysregulation, characterized by autoreactive lymphocytes penetrating the BBB, resulting in an inflammatory cascade that leads to demyelination, axonal transection, and neurologic deficits (240). The entry of lymphocytes into the CNS requires transmigration through the inflamed endothelium, and the prevention of this process should provide anti-inflammatory therapy in MS (241). Natalizumab was the first monoclonal antibody approved in 2004 for the treatment of RRMS. It is a humanized recombinant IgG4 monoclonal antibody that inhibits leukocyte extravasation into the CNS and intestinal tract by blocking the α4 subunit of integrin molecules on leukocytes (242). Integrins are cell-surface glycoproteins that facilitate cell-matrix adhesion and mediate leukocyte rolling and adhesion to the endothelium prior to extravasation (243). By inhibiting their interaction with vascular cell-adhesion molecule (VCAM)-1 expressed on endothelial cells, natalizumab prevents T lymphocytes from crossing the BBB, thereby reducing inflammation in the brain-tissue compartment (244). In 1992, a study by Yednock et al. concluded that antibodies against the α4-integrin substantially restricted the accumulation of leukocytes in the CNS and prevented the development of a model mimicking MS in rats known as experimental autoimmune encephalomyelitis (EAE) (245). These findings paved the way for early clinical trials with natalizumab. Perhaps equally important, natalizumab was later shown to sequester T cell and B cell subsets out of the CNS compartment (246–248), providing proof-of-concept evidence that a reduction in adaptive immune-cell access to the CNS benefits RRMS patients.

The efficacy of natalizumab for treating RRMS was shown in two phase-III trials: the AFFIRM and SENTINEL studies (249), In the AFFIRM study, 942 patients with RRMS were enrolled in a 2:1 ratio to receive natalizumab 300 mg every 4 weeks or a placebo. The primary outcomes included the clinical-relapse rate at 1 year and the sustained disability-progression rate at 2 years. The results showed that natalizumab reduced the ARR by 68% and lowered the risk of sustained disability progression at 2 years by 42% (249). The SENTINEL study enrolled 1,171 patients who had at least one relapse whilst on IFNβ-1a therapy in the previous 12 months. They received intramuscular IFNβ-1a in combination with 300 mg of natalizumab or a placebo. The outcome measures were identical to those of the AFFIRM study and showed that combination therapy with natalizumab yielded a 55% reduction in the ARR and a 24% reduction in the risk of sustained disability progression at 2 years (250). Both studies also showed significant reductions in the number of new or enlarging T2 lesions and enhancing lesions on MRI in patients receiving natalizumab. Natalizumab was studied further in patients with SPMS in the phase-III ASCEND trial, which did not meet the primary endpoint of disability progression (251), although its target α4-integrin is highly expressed in active lesions of patients who died in late secondary progressive MS (252). These results highlight the different disease pathophysiology that drives progressive forms of MS, which is characterized by axonal/neurodegeneration, innate immune responses mediated by CNS resident cells, development of meningeal lymphoid follicles contributing to expanding number of cortical lesions, and compartmentalized inflammation (137, 253).

Shortly after natalizumab was approved in 2004, the drug was withdrawn from the market after three patients developed PML, a life-threatening CNS-demyelinating disease caused by infection of oligodendrocytes with the John Cunningham virus (JCV). Natalizumab associated PML carries an average mortality rate of 23%, and survivors often develop debilitating neurological deficits from the disease and its treatment sequelae (254). In immunocompetent individuals, JCV almost never causes disease and remains latent in more than half of the world's population (255). PML more frequently affects immunosuppressed individuals, such as patients with acquired immune deficiency syndrome (AIDS), however, in patients taking natalizumab who are not systemically immunosuppressed, the disease is thought to be caused by forced migration of cells harboring JCV out of the bone marrow and the upregulation of gene products in B cell maturation that also promote JCV growth (256). For patients with a suspected PML diagnosis, natalizumab must be discontinued, and treatment with plasma exchange or immunoadsorption can help eliminate remaining circulating natalizumab (257). Patients might develop paradoxically worsening neurologic deficits due to an overwhelming inflammatory reaction due to the recovering immune system, a condition known as immune reconstitution inflammatory syndrome (IRIS), for which a corticosteroid can be given to provide modest benefits (258).

In 2006, natalizumab was reintroduced to the market with a black-box warning about PML risks. Prescribers and patients are required to enroll in a program that the FDA developed, known as Tysabri Outreach Unified Commitment to Health (TOUCH), which informs providers and patients about PML risks. The risks of developing PML have been identified in post-marketing analysis and include the presence of anti-JCV antibodies, prior exposure to immunosuppressants, and more than 2 years of natalizumab therapy (259, 260). In the absence of anti-JCV antibodies, the risk for PML is <0.1/1,000, but the risk increases up to 23/1,000 in patients with all three risk factors (261). Testing for JCV serology is recommended every 6 months for patients with negative and indeterminate results, given a seroconversion rate of 8.5–10.3% per year in natalizumab treated patients (262). Routine surveillance MRIs also can detect early stages of the disease (263, 264). Since there are no guidelines for quantifying the risk of PML, it is up to the clinician and the patient to be aware of risk factors and consider switching therapies based on the risks and benefits from continuing the drug.

Anti-idiotypic antibodies against natalizumab are known to reduce the clinical efficacy, as well as to increase the likelihood of infusion-related adverse events (265). The presence of anti-natalizumab antibodies may be transient or persistently positive, defined as present on at least two occasions 6 weeks apart (249). In the AFFIRM study, antibodies against natalizumab were detected in 9% of patients on natalizumab (6% persistently positive), and in the SENTINEL study, they were present in 12% of patients on natalizumab and IFNβ-1a (6% persistently positive). It is recommended that patients with ongoing disease activity or persistent adverse infusion reactions be tested for antibodies against natalizumab (265, 266). Natalizumab should not be given during pregnancy. However, available data from reports do not show a significant increase in abnormalities compared to other MS patients, and it might be administered based on the individual case. An individual plan should to be made for each female patient who wishes to conceive (38).

The risk of rebound MS disease activity exists for patients who discontinue natalizumab for reasons such as the increased risk of PML, pregnancy, or the presence of neutralizing antibodies. After cessation of natalizumab, it takes 3 months for serum natalizumab concentrations to fall below the threshold of 1 μg/mL for α4-integrin desaturation (267), and CSF lymphocyte counts remain suppressed for up to 6 months (246). Correlating with these laboratory findings, disease recurrence typically starts from 2 to 6 months after natalizumab discontinuation and peaks at 5–8 months (268). Clinical worsening of MS is seen in 20–30% of patients who stop taking natalizumab, and 50–60% show worsening MRI lesions with either new gadolinium-enhancing lesions or new or enlarging T2-hyperintense lesions (268). Restarting therapy with alternative agents—including GA, IFN-ß, and fingolimod—cannot completely prevent recurrence of disease, however, patients who have switched therapies generally have lower rebound activity (269–275). In addition, shortening the natalizumab washout period and tapering cessation have been shown to reduce the risk of rebound disease further (276–278).

Immunologically, disease reactivation after cessation of natalizumab has been associated with some degree of immune reconstitution in the CNS.

Table 4 summarizes proposed modes of action of all approved MS therapeutics.

**Table 4:** Overview on supposed modes of action of approved therapeutics in MS and its proposed effects on the immune system.

**Substance**	**Administration**	**Mode of action**	**Effects on immune System**
IFN-ß	SC, IM	Not elucidated in detail part of the type I interferon class (activation of JAK/STAT pathways)	pro-inflammatory lymphocyte activation ↓; anti-inflammatory lymphocyte activation↑; TH1 → TH2 shift; lymphocyte migration into CNS↓; monocyte activation↓
GA	SC	Not elucidated in detail variety of immunological and non-immunological pathways Competition with myelin antigens for MHC binding site on APCs	T cell autoreactivity to myelin antigens ↓; generation of GA-reactive TH2 cells; TH1 → TH2 shift; Tregs ↑; number of B cells, plasmablasts and memory B cells↓; shift from pro-inflammatory to anti-inflammatory B cell phenotypes
S1P	PO	functional antagonist of S1PR egress of lymphocytes from lymph nodes↓; effects on neuronal and glial cells in CNS	lymphocyte egression ↓; cytotoxicity ↓; regulatory T cells↑
MTX	IV	type II topoisomerase inhibitor induction of cell lysis and initiation of programmed cell death on B cells and T cells	Levels of T cells and B cells↓; effects on innate immune system (macrophage proliferation) ↓; antigen presentation↓; antibody production↓; pro-inflammatory cytokine secretion↓
TERI	PO	Inhibition of DHODH → reduction in de-novo pyrimidine synthesis and DNA replication of highly proliferating T cells and B cells↓	Activated T cell and B cell proliferation ↓; Tregs↑; pro-inflammatory cytokines ↓
DMF	PO	activation of Nrf-2 pathway inhibition of NF-κB pathway activation of HCAR2	Nrf2↑; Tregs and CD56bright NK-cells↑; antioxidant proteins ↑; BBB migration ↓; TH1/TH17 → TH2 shift; pro-inflammatory cytokines ↓; apoptosis of T and B cells↑; Shift from pro-inflammatory to anti-inflammatory microglia
CLAD	PO	Purine nucleoside analog that interferes with DNA synthesis and repair, preferentially in activated lymphocytes	Lymphocytes ↓, relative increase in regulatory T cells
ALT	IV	mAb (IgG1) targeting CD52 predominantly on T cells and B cells, leading to cells lysis via CDC and ADCC	T cells and B cells ↓; CD56bright NK and Tregs↑; remodeling of lymphocytes
OCR	IV	mAb (IgG1) targeting CD20 on immature and mature B cells leading to cells lysis via ADCC > CDC	B cell depletion; regulatory B cells↑
NTZ	IV	mAb (IgG4) targeting and inbiting α4 subunit of integrin molecules on leukocytes;	lymphocyte migration into CNS↓

*IFN-, interferon beta; GA, glatirameracetate; S1P, sphingosine-1-phosphat receptor modulator (fingolimod, siponimod); MTX, mitoxantrone; TERI, teriflunomide; DMF, dimethylfumarate; CLAD, cladribine; ALT, alemtuzumab; OCR, ocrelizumab; NTZ, natalizumab; IM, intramuscularly; IV, intravenously; PO, orally; SC, subcutaneously; ADCC, antibody-dependent, cell-mediated cytolysis; CDC, complement-dependent cytolysis; DHODH, dihydroorotate dehydrogenase; HCAR2, hydroxycarboxylic acid receptor 2; mAb, monoclonal antibody; MBP, myelin basic protein; MMF, mono-methyl fumarate*.

## Conclusion

The widening of the MS treatment landscape over the past few decades mirrors progress in our understanding of MS pathophysiology. Although the mode of action is not known in detail for some of the approved treatments, the known mechanisms for other agents support our current concepts of the pathophysiology of early MS. Moreover, some treatments, such as ocrelizumab, have broadened our perspective on pathogenic MS events (230), highlighting the importance of B cells in treating MS (279).

As stated, the progress that has been achieved over the past few decades regarding MS pharmacotherapies is tremendous, and perhaps unprecedented for any medical subspecialty. In addition to benefitting patients with RRMS and PPMS, many of the agents currently approved for MS patients have informed us about relevant molecular and cellular targets in this disorder. However, challenges remain, including the potentially serious AEs. Long-term safety data is needed to detect rare but serious AEs. Based on these data monitoring must be adapted to decrease the risk of severe or even life-threatening events (as JCV-Abs for PML in patients treated with natalizumab or secondary autoimmunities in alemtuzumab treated patients). Pharmacovigilance and a better understanding of the host factors that lead to these adverse events hopefully will reduce their incidence. MS is a heterogenous disease and the individual disease progression and prognosis is not predictable. Understanding the mode of action of the various treatment options is of utmost importance to (I) foresee side effects, and (II) to define the best possible treatment, especially for patients that have not responded to treatments. Further treatments such as autologous haematopoietic-stem-cell transplantation (aHCST) are assessed in trials (280). aHCST is not approved but shows promising results. Risks and benefits for patients must be balanced, and again the appropriate patient selection is of utmost importance.

Finally, there is no evidence that any of the currently approved agents benefit patients with non-active MS (281). Currently there is no agreement when to stop or de-escalate treatment. Re-activation of disease might be a risk (282, 283). Further studies are ongoing or needed. The definition of active and non-active MS needs to be improved by appropriate biomarkers to prevent patients from being treated with agents that provide no benefit to them yet expose them to potential AEs.

## Author Contributions

All authors contributed at least one chapter to this review. All authors reviewed for intellectual content.

### Conflict of Interest Statement

PR received honoraries from consultancy or lectures from AbbVie, Biogen, Celgene (institutional), Merck, Roche, Sanofi Genzyme, Teva. He served on advisory boards for Biogen, Merck, Roche, Sandoz. PR received research support from Biogen, Merck Serono and Roche. RM received honoraria/consulting fees: Actelion, Bayer Healthcare, Biogen-Idec, Medison, Merck-Serono, Neopharm, Novartis, Roche, Sanofi-Genzyme, Teva, and TG-Therapeutics. He served on advisory boards: Bayer Healthcare, Genzyme, Medison, Merck, Neopharm, Novartis, Roche Teva, and TG-Therapeutics. He received research grants: Bayer Healthcare, Medison, Merck-Serono, Novartis, and Teva. MH is on the steering committee for the Novartis FLUENT study. JS received personal compensation as a speaker or consultant from Bayer, Biogen, Merck, Gerot-Lannach, Roche, Sanofi, and Teva. He is member of advisory boards of Biogen, Merck, Sanofi, Roche, and MedDay. His institution received unrestricted grants from Biogen, Merck, Roche, and Sanofi. ZI has served on scientific advisory board for Biogen, Sanofi-Genzyme, Teva, Roche, Novartis, Merck; has received honoraria for lecturing from Biogen, Merck, Teva, Novartis, Sanofi-Genzyme; research support from Biogen, Sanofi-Genzyme, Merck; and support for congress participation from Biogen, Sanofi-Genzyme, Teva, Roche, Novartis, Merck. CW received speaker honoraria (institutional only) and/or research funding from Biogen, Novartis, Sanofi-Genzyme, and Roche. The remaining authors declare that the research was conducted in the absence of any commercial or financial relationships that could be construed as a potential conflict of interest.

## Footnotes

^1^Commissioner O of the. *FDA Expands Approval of Gilenya to Treat Multiple Sclerosis in Pediatric Patients [Internet]*. FDA (2018). Available online at: http://www.fda.gov/news-events/press-announcements/fda-expands-approval-gilenya-treat-multiple-sclerosis-pediatric-patients (accessed June 13, 2019).

^2^Fingolimod (Gilenya?): bradycardia and heart block [Internet]. GOV.UK. Available online at: https://www.gov.uk/drug-safety-update/fingolimod-gilenya-bradycardia-and-heart-block (accessed May 30, 2019).

^3^Commissioner O of the. Press Announcements - FDA approves new oral drug to treat multiple sclerosis [Internet]. Available online at: https://www.fda.gov/NewsEvents/Newsroom/PressAnnouncements/ucm634469.htm (accessed April 7, 2019).

^4^novantrone-article-30-referral-annex-iii_en.pdf [Internet]. Available online at: https://www.ema.europa.eu/en/documents/referral/novantrone-article-30-referral-annex-iii_en.pdf (accessed April 7, 2019).

^5^ml-concentrate-solution-infusion_de.pdf [Internet]. Available online at: https://www.ema.europa.eu/en/documents/referral/questions-answers-novantrone-associated-names-mitoxantrone-2-mg/ml-concentrate-solution-infusion_de.pdf (accessed April 7, 2019).

^6^Foechterlen D. oder an das Bundesinstitut für Arzneimittel und Medizinprodukte (BfArM): elektronisch über das Internet www.bfarm.de – Arzneimittel – Pharmakovigilanz – Risiken melden; oder schriftlich an die Postadresse Kurt-Georg-Kiesinger-Allee 3, 53175 Bonn, oder per Fax 0228-207 5207.2.

^7^Commissioner O of the. Press Announcements - FDA approves new oral treatment for multiple sclerosis [Internet]. Available online at: https://www.fda.gov/NewsEvents/Newsroom/PressAnnouncements/ucm634837.htm (accessed April 7, 2019).

^8^WC500234561.pdf [Internet]. Available online at: http://www.ema.europa.eu/docs/en_GB/document_library/EPAR_-_Product_Information/human/004230/WC500234561.pdf (cited September 26, 2018).

^9^LEMTRADA® (alemtuzumab) | Official Healthcare Professional Site [Internet]. Available online at: https://www.lemtradahcp.com/safety-information (accesed June 2, 2019).

^10^ocrevus-epar-product-information_en.pdf [Internet]. Available online at: https://www.ema.europa.eu/en/documents/product-information/ocrevus- epar-product-information_en.pdf (accessed June 1, 2019).

^11^OCREVUS safely and effectively, 18.
